# Navigating Epstein–Barr Virus (EBV) and Post-Transplant Lymphoproliferative Disorder (PTLD) in Pediatric Liver Transplantation: Current Knowledge and Strategies for Treatment and Surveillance

**DOI:** 10.3390/v17020254

**Published:** 2025-02-13

**Authors:** Erin Y. Chen, Natasha Dilwali, Krupa R. Mysore, Sara Hassan, Sara Kathryn Smith, Wikrom Karnsakul

**Affiliations:** 1 School of Medicine, The Johns Hopkins University, Baltimore, MD 21205, USA; echen41@jh.edu; 2 Division of Pediatric Gastroenterology, Hepatology, and Nutrition, Department of Pediatrics, Johns Hopkins Children’s Center, Baltimore, MD 21287, USA; ssmit403@jhmi.edu (S.K.S.); wkarnsa1@jhmi.edu (W.K.); 3 Division of Gastroenterology, Hepatology, and Nutrition, Department of Pediatrics, Baylor College of Medicine and Texas Children’s Hospital, Houston, TX 77030, USA; mysore@bcm.edu; 4 Division of Pediatric Gastroenterology, Hepatology, and Nutrition, Department of Pediatrics, UT Southwestern, Children’s Medical Center of Dallas, Dallas, TX 75235, USA; sara.hassan@utsouthwestern.edu

**Keywords:** EBV, PTLD, post-transplant lymphoproliferative disorder, pediatric liver transplant

## Abstract

Epstein–Barr virus (EBV) is strongly associated with the development of post-transplant lymphoproliferative disorder (PTLD) in pediatric liver transplant recipients. PTLD is one of the most common malignancies following liver transplantation and is associated with significant morbidity and mortality. Factors such as EBV–serostatus mismatch and prolonged or high levels of immunosuppression impact a patient’s risk of developing PTLD. While pre-transplant EBV serological screening and post-transplant monitoring of EBV-DNA levels are strongly recommended, universal guidelines for its prevention and management are lacking. Due to a lack of robust prospective studies, current clinical practices vary widely. The treatment of PTLD typically involves reducing immunosuppression and using targeted therapies such as rituximab, or chemotherapy for refractory cases. This review aims to address our current understanding of EBV’s relationship with PTLD, evaluate the available treatment modalities, and highlight evolving strategies for using EBV as a biomarker for PTLD screening and prevention.

## 1. Introduction

### 1.1. What Is EBV

Epstein–Barr virus (EBV) is a double-stranded linear DNA virus in the Herpesviridae family that infects over 90% of adults worldwide [1,2,3]. EBV infection often occurs in childhood and is usually asymptomatic or may present as infectious mononucleosis [2,3]. Beyond acute infection, EBV is implicated in a range of diseases, including multiple sclerosis, Burkitt lymphoma, Hodgkin lymphoma, nasopharyngeal carcinoma, and post-transplant lymphoproliferative disorder (PTLD) [3,4,5,6,7,8].

In transplant recipients, EBV plays a critical role in the pathogenesis of PTLD by driving uncontrolled B-cell proliferation, often due to inadequate T-cell-mediated immune surveillance [9,10,11,12,13]. The risk of PTLD secondary to EBV is higher in pediatric patients. One study showed that 98% of pediatric PTLD cases were EBV-positive compared to 68% of adult PTLD cases [14]. In another study, pediatric patients with a post-liver-transplant primary EBV infection were found to be 17 times more likely to develop PTLD than those who had pre-transplant serologies positive for EBV [15].

The life cycle of EBV involves three phases: primary infection, latency, and reactivation. Primary infection occurs via the oropharyngeal epithelium, spreading to naive B-cells [16]. After the initial infection, EBV persists within memory B-cells, which can harbor the virus throughout a person’s life [2,12,13,16]. During latency, EBV expresses latency-associated proteins (e.g., LMP1, LMP2) and EBV nuclear antigens (EBNAs) which facilitate immune evasion, viral replication, and host–cell survival [17,18]. Latency-associated non-coding RNAs, including Epstein–Barr virus-encoded small RNAs (EBERs) and BAMIII fragment A rightward transcripts (BARTs), further contribute to immune modulation and cell survival [2,17]. The reactivation of EBV, typically under immunosuppressive conditions, allows the virus to re-enter its lytic phase, producing new virions and potentially driving PTLD [2,12,13,16].

### 1.2. How EBV Is Detected Currently?

EBV is detected through antibody serology, nucleic acid amplification testing (NAAT), and EBER flow fluorescence in situ hybridization (EBER flow FISH). During infection, the immune system produces antibodies to its viral components. These antibodies can be detected on serologic assays in immunocompetent hosts and can indicate the temporality of the EBV infection. However, serologic assays are unreliable in immunosuppressed patients due to their altered antibody responses. Studies have shown that immunosuppressed children who had negative EBV serologies often had low-to-undetectable EBV antibody titers after infection. Meanwhile, children who had positive EBV serologies who were then immunosuppressed after transplantation tended to have high EBV antibody titers [12,18,19].

EBV DNAemia can be measured through NAAT. In immunocompetent hosts, EBV DNAemia is often low or undetectable. During times of immune disruption or immunosuppression, these levels can transiently rise and become detectable with NAAT, making surveillance crucial. However, the quantification of the EBV viral load can vary greatly, depending on what is being measured (whole blood vs. plasma) and the quality of the NAAT assay [12,20,21]. Per the International Pediatric Transplant Association (IPTA) guidelines, either whole blood or plasma can be used, each with its own potential benefits, as whole blood is more sensitive, while plasma is more specific [20,21]. EBER flow FISH, using EBV-encoded small RNAs (EBERs), is an emerging tool that identifies infected cells in both their lytic and latent stages. Unlike PCR, it can differentiate EBV infections across various cell types, offering insights into the pathogenesis and disease mechanisms of EBV (Table 1) [20,21,22,23,24,25].

## 2. Post-Transplant Lymphoproliferative Disorder (PTLD)

### 2.1. Why Does It Matter in Pediatric Solid Organ Transplants?

PTLD accounts for approximately 70% of all malignancies diagnosed following SOT in pediatric patients and occurs in up to 20% of cases, with mortality rates reaching 50% depending on the transplanted organ [12,13,26,27]. In pediatric liver transplantation, its prevalence is approximately 2–8%, with a mortality of 44% [12,28]. The outcomes in SOT recipients are less favorable due to the limited effectiveness of preemptive EBV therapies compared to hematopoietic stem cell transplant (HSCT) settings [26,29]. Beyond mortality, PTLD impacts long-term SOT outcomes, serving as a risk factor for chronic allograft rejection and graft loss, often necessitating re-transplantation [30].

### 2.2. Clinical Presentation

**Figure 1:**
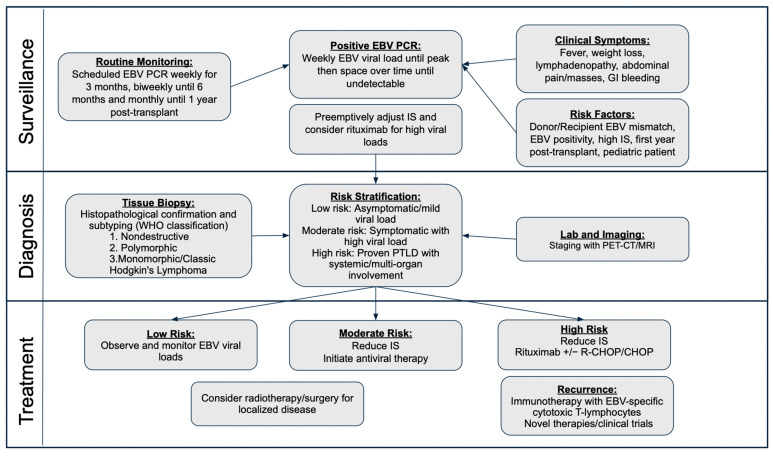
Algorithm for EBV surveillance, diagnosis and management.

The clinical presentation and symptoms of PTLD are highly variable and non-specific (Figure 1). Patients may be asymptomatic or present with fever, malaise, weight loss, anorexia, diarrhea, and night sweats. Some children present with classic infectious mononucleosis symptoms and lymphadenopathy. PTLD can present with focal symptoms related to the affected organ or, rarely, with disseminated multiorgan disease that resembles sepsis. Patients may experience a sore throat, voice changes, snoring, cough, or trouble breathing if tonsillar lymph nodes are affected [31,32,33]. Gastrointestinal involvement is common, presenting with hepatosplenomegaly, gastrointestinal bleeding, and abdominal pain [34,35]. While rare, PTLD can sometimes involve the central nervous system and cause focal neurologic deficits [35,36,37]. PTLD of the allograft organ can also occur, and acute organ rejection is often mistaken for PTLD. About 33% of pediatric liver transplant PTLD manifestations involve the liver allograft [12,38]. Overall, children more commonly present with isolated cervical lymphadenopathy, intussusception, mesenteric adenopathy, and bowel wall thickening compared to adults, who are more likely to have extra-nodal involvement of the gastrointestinal tract, allograft, and central nervous system [39]. PTLD presents with a bimodal distribution, with most cases occurring within one year of transplantation, and the rest presenting five to ten years after transplantation [40]. Progression can vary widely, from a rapidly advancing disease to prolonged, fluctuating courses.

### 2.3. Diagnosis

The definitive diagnosis of PTLD relies on a tissue biopsy, which allows for its histological evaluation and classification. Under the 2017 WHO guidelines, PTLD was categorized into four types: non-destructive, polymorphic, monomorphic, and classic Hodgkin lymphoma [41,42]. In 2022, the WHO updated this classification to emphasize different forms of immune dysfunction, aligning more closely with clinical scenarios and patient management (Table 2) [10,43,44]. Comprehensive laboratory and imaging studies are crucial in supporting its diagnosis and staging. The initial workup often includes a complete blood count, complete metabolic panel, lactate dehydrogenase, uric acid, EBV PCR in previously EBV-negative patients, and EBV serologies. If the concern about PTLD is high, computed tomography (CT) or magnetic resonance imaging (MRI) with contrast is obtained to localize the areas of involvement and stage the disease. PET-CT is particularly valuable for detecting metabolically active lesions and staging [13,43,45]. Staging is based on current defined guidelines for staging lymphoma [46]. If gastrointestinal symptoms are present, a gastrointestinal endoscopy is often performed to localize and biopsy the potential lesion [13,34,35]. The prompt identification and classification of PTLD are critical for guiding timely, targeted treatment to improve outcomes (Figure 1).

## 3. The Connection Between EBV and PTLD

### Epidemiology

The rising incidence of PTLD can be attributed to multiple factors. One of the primary reasons is the increasing number of SOTs performed, particularly liver transplants (LTs), which inherently increases the pool of individuals at risk for PTLD. Additionally, there has been a growing awareness about PTLD, leading to more vigilant monitoring and recognition of the condition. Furthermore, advancements in diagnostic techniques, including more sensitive imaging modalities and molecular testing, have improved our ability to detect PTLD at earlier stages or in less obvious clinical presentations, contributing to the observed increase in reported cases [47].

The prevalence of PTLD in LT recipients is impacted by several factors, resulting in a 2–12% incidence rate. One of the most significant factors is the immunosuppressive regimen used to prevent organ rejection, as more intense or prolonged immunosuppression can increase the risk of a patient developing PTLD. Additionally, the recipient’s EBV serostatus plays a critical role, as those who are EBV-negative at the time of transplant are at a higher risk when exposed to the virus post-transplant. Furthermore, EBV donor–recipient mismatch significantly elevates risk, particularly when an EBV-positive donor transmits the virus to an EBV-negative recipient [15,47,48,49].

## 4. Pathophysiology

Approximately 55–75% of PTLDs are associated with an EBV infection linked to a primary infection, reactivation, or donor transmission [10]. EBV reactivation can occur due to a variety of factors. The use of immunosuppressant mycophenolate mofetil (MMF) post-transplant has been shown to reduce Vγ2+ T cell proliferation, a T-cell critical for both innate and adaptive immunity against EBV-infected cells [2,50,51]. EBV reactivation is also frequently observed in co-infections such as cytomegalovirus, syphilis, human papillomavirus, Kaposi sarcoma-associated herpesvirus, and COVID-19 [2,52]. Additionally, non-infectious factors such as radiation, oxidative and cellular stress, B-cell maturation, and psychological stress have been linked to increased EBV viral load and reactivation [2].

While the precise mechanisms at play remain unclear, the EBV genome is found in >90% of B-cell PTLD cases occurring within the first year after a SOT [11,48]. The host immune system typically regulates EBV-infected latent cell proliferation and viral reactivation through mechanisms such as cytotoxic T lymphocytes (CTLs), which target EBV antigens like LMP1 and EBNA2 [9,12,16,53]. EBV latent membrane proteins 1 and 2a (LMP-1 and LMP-2a) mimic CD40 and B-cell receptor growth signals, respectively, providing survival signals to B-cells. CD40 activation inhibits viral lytic infection, while interleukin-10 secretion by EBV stimulates B-cell proliferation [10]. These processes, combined with EBV oncogene expression, immunosuppression, chronic antigen exposure, and impaired T-cell surveillance, contribute to B-cell transformation, proliferation, and apoptosis inhibition, driving PTLD development [48,54].

## 5. Donor/Recipient Status

Donor-transmitted EBV infections are particularly common in EBV-mismatched patients (donor seropositive/recipient seronegative), especially in children who are often EBV-naïve [48]. Monitoring recipients’ EBV serological status and EBV DNAemia post-transplant has proven effective in stratifying EBV risk. For instance, Bernabeu et al. found that approximately 20% of asymptomatic post-transplant children carried high EBV loads for months to years following a primary infection [15,55].

## 6. EBV-Positive vs. EBV-Negative PTLD

In a large analysis of over 160,000 pediatric and adult liver transplant recipients, Dharnidharka et al. highlighted an elevated PTLD risk in EBV-seronegative individuals [48,56]. Early-onset PTLDs are predominantly EBV-positive and present within the first-year post-transplant, while EBV-negative PTLDs typically manifest 5–10 years later. EBV-positive PTLD also tends to present with a polymorphic histology, while EBV-negative PTLD is frequently monomorphic and resembles the lymphomas found in immunocompetent hosts [57].

Although EBV plays a significant role, the pathogenesis of PTLD also involves genetic alterations, such as chromosomal translocations, gene mutations, and epigenetic changes. Further, other viral infections (e.g., human herpes virus 5, 6, and 8), hit-and-run EBV infections, and chronic antigenic stimulation by the allograft, including antibody-mediated rejection, may contribute to EBV-negative PTLD, although this remains unconfirmed [57,58].

## 7. Treatment of PTLD

**Figure 2:**
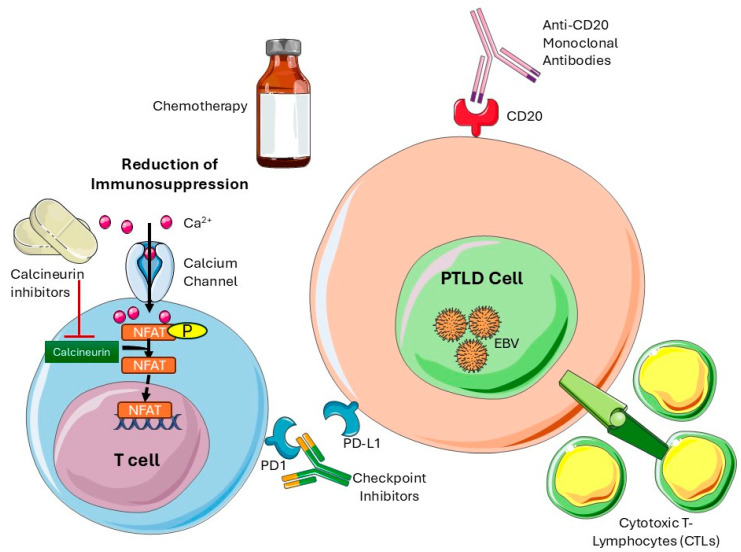
Treatment strategies for EBV PTLD vary and may include a reduction in immunosuppression, the use of chemotherapy agents, anti-CD20 monoclonal antibodies, and cytotoxic T lymphocytes. EBV: Epstein–Barr virus; PTLD: post-transplant lymphoproliferative disorder. (This schematic was created using Servier Medical Art templates, provided by Servier, licensed under a Creative Commons Attribution 4.0 international license; https://smart.servier.com).

The goal of PTLD treatment is to achieve remission while preserving the allograft. Due to the rarity of PTLD, most evidence is derived from retrospective studies [59]. First-line management includes reducing immunosuppression (RIS), followed by rituximab (anti-CD20 monoclonal antibody) and, in some cases, chemotherapy (Figure 2; Table 3). Surgery and radiation are limited to isolated or palliative cases [39]. Novel therapies including immunotherapy, cytokine treatment, and anti-EBV-based therapy continue to be explored (Figure 2; Table 3). Currently, there are no standardized treatment regimens for PTLD. Therapy should be individualized based on the stage and type of PTLD. Multidisciplinary approaches with the early involvement of oncology are essential for the management of PTLD.

1. Reducing Immunosuppression (RIS):

RIS aims to restore recipient immunity by enabling EBV-specific T-lymphocyte proliferation [60]. Early, low-burden, EBV-driven cases respond best to RIS, but outcomes are less favorable in aggressive disease or late-onset PTLD [56]. The risks include a delayed response (3–5 weeks), graft rejection, and organ failure [59]. Protocols vary by disease severity and transplant center, often involving calcineurin inhibitor reduction and antimetabolite discontinuation [54]. In pediatric solid organ transplant patients, predictors for a poor response to RIS alone include CD20 or EBV negativity, late-onset PTLD, or neural involvement [39,61,62].

2. Rituximab:

Rituximab is a chimeric murine and human monoclonal anti-CD20 antibody. The CD20 antigen is found on B-cell lymphocytes, where rituximab can bind and mediate B-cell lysis and CD20+ tumors. It is typically used after RIS failure or as part of sequential therapy with chemotherapy [61,63]. As with RIS, monotherapy with rituximab has not shown a long-term event-free survival—in a combined analysis of two prospective trials of rituximab monotherapy in PTLD, 26% of complete and partial responders required further therapy within 1 year [63,64,65]. Recent data have shown that the early introduction of rituximab in PTLD improves outcomes, with a reported overall survival of 73% compared to 33% in those who received frontline rituximab treatment [65].

3. Chemotherapy:

Historically, chemotherapy used for lymphoproliferative disorders has not been used in SOT recipients due to its high treatment-related mortality (TRM) and the risk of graft rejection [62]. However, those who can tolerate it can achieve lasting remission from PTLD [39,61]. The PTLD-1 trial established that 20% of patients achieved complete remission with monotherapy and 57% achieved complete remission with sequential rituximab and CHOP chemotherapy for aggressive cases [66,67]. In terms of pediatric PTLD, a pilot phase 2 trial was carried out with the Children’s Oncology Group, with a two-year event-free survival rate of 77% and an overall survival rate of 80% found [68]. If chemotherapy is utilized, supportive measures such as prophylactic antibodies, continued RIS, and the use of growth factors should be considered [62].

4. Novel Therapies:

The treatment of PTLD in transplant recipients generates special challenges due to organ dysfunction with the long-term use of calcineurin inhibitors, concerns about graft function, and the potential for toxicity and higher infection risk. These issues have led to the consideration of novel treatment options [61]:

A. CD30 Targeting: The CD30 antigen is an activation marker expressed by activated B and T lymphocytes, recently found to be commonly expressed in all types of PTLD. Brentuximab vedotin, an anti-CD30 antibody–drug conjugate, shows promise in CD30-positive PTLD when administered concurrently with rituximab [69,70,71].
B. T Cell Therapy: Donor-derived EBV-specific cytotoxic T-lymphocyte (CTL) infusions have shown efficacy in solid organ transplant recipients, with response rates exceeding 50%. Due to immune suppression in transplant recipients, the cytotoxic T-cell response is poor in PTLD. Donor-derived anti-EBV cytotoxic T lymphocytes (CTL) can be infused into a patient, altering their EBV-specific cellular immune response and leading to PTLD regression [72]. In SOT, CTL infusions have achieved overall response rates of 64% at five weeks and 52% at six months [72]. In an ongoing phase 3 trial (ALLELE), the allogenic EBV-CTL treatment tabelecleucel showed an objective response in 52% of SOT patients with relapsed or refractory PTLD without significant treatment-related adverse events [73].
C. Interleukin-6: Anti-IL-6 monoclonal antibodies have led to partial or complete remission in small cohorts. IL-6 promotes the growth of EBV-infected B-cells and patients with PTLD have high levels of IL-6. Based on this, anti-IL6 monoclonal antibodies have been trialed, which led to complete remission in 45% and partial remission in 25% of the 12 patients [74]. However, the data are limited, and further study is needed.

**Table 3:** Treatment regimens of PTLD.

Treatment	Dosing	Risks
**Reduction in Immunosuppression**	**N/A**	Delayed response (3–5 wks), graft rejection, organ failure [62]
**Rituximab (monotherapy)**	Four intravenous (IV) infusions, each of 375 mg/m^2^, on days 1, 8, 15, and 22 of treatment [63]	Severe infection, neutropenia, hypogammaglobulinemia
**Chemotherapy (CHOP) w/Rituximab**	Six cycles, 3 weeks apart Cycles 1 and 2: *Cyclophosphamide* (600 mg/m^2^ intravenous) day 1 *prednisone* (1 mg/kg orally twice a day) or *methylprednisolone* (0.8 mg/kg intravenous every 12 h) on days 1–5, and *rituximab* (375 mg/m^2^ intravenous) on days 1, 8 and 15 Cycles 3–6: same as above, without rituximab [68]	Treatment-related mortality, graft rejection [68]
**Targeting CD30 (Brentuximab vedotin) w/Rituximab**	Induction: *Rituximab* 375 mg/m^2^ given on days 1, 8, 15, and 22 of treatment *Brentuximab vedotin* 1.2 mg/kg given on days 1, 8, and 15 Maintenance: *Rituximab* 375 mg/m^2^ every 6 wks *Brentuximab vedotin* 1.8 mg/kg every 3 weeks [71]	Treatment-related toxicity—infection, febrile neutropenia, peripheral neuropathy, pancreatitis [71]
**T-Cell Therapy (e.g., tabelecleucel)**	Weekly intravenous infusions of CTLs (2 × 10^6^ CTLs/kg) for total of 4 weeks [72,73]	Cytokine release syndrome, infusion toxicity [73,75]
**Anti-Interleukin-6 (IL-6) Antibodies**	Up to 0.8 mg/kg per day for 15 days [74]	Infection, thrombocytopenia, neutropenia, dyslipidemia, elevated liver enzymes [76]

CHOP = cyclophosphamide, doxorubicin (hydroxydaunorubicin), vincristine (Oncovin), and prednisone; CTL = Cytotoxic T-Lymphocytes.

## 8. Screening and Surveillance

Pre-transplant EBV serological screening of both the donor and recipient is critical for risk stratification. Most commonly seen post-transplant, EBV DNAemia is monitored using PCR assays of whole blood or plasma, though practices vary globally. Unfortunately, there are no specific criteria used to define EBV DNAemia cut off values to start preemptive management, as EBV levels at PTLD diagnosis are quite variable. Sustained high viral loads are associated with increased PTLD risk and hence serial monitoring is advocated for [15,77,78,79]. Since the risk of PTLD has been linked to immunosuppression use, and its incidence is highest in the first year post-transplantation, transplant centers generally screen for EBV titers in the blood or plasma more frequently during this period [80]. The International Pediatric Transplant Association (IPTA) recently proposed surveillance guidelines in 2023. For EBV-negative patients who receive an organ from EBV-positive donors, they propose weekly EBV PCR surveillance for three months post-transplant, followed by biweekly surveillance until six months, and then monthly until one year. If their EBV PCR becomes positive, then weekly EBV viral load monitoring should be performed until the viral load peaks and begins down-trending after two–four weeks. EBV viral load monitoring is then adjusted to biweekly, monthly, and then every three months until the viral load is undetectable [21]. A younger age at transplant and seronegativity pre-transplant are risk factors for the development of early PTLD [15]. Though universal cutoff values remain undefined, centers commonly preemptively adjust immunosuppression and use rituximab for high EBV loads [48,80,81]. Standardized algorithms for EBV monitoring, risk-based immunosuppression reduction, and preemptive rituximab use are needed in pediatric liver transplantation.

## 9. Conclusions

PTLD is a rising concern in pediatric liver transplantation and is strongly associated with EBV infection. Effective prevention and treatment strategies, including immunosuppression management, rituximab, and novel therapies, continue to evolve. However, systematic EBV monitoring and risk-based interventions remain critical in mitigating the incidence of PTLD and improving outcomes. Further research is mandatory to determine the best management strategies for children found to be at higher risk for PTLD, how to approach post-transplant patients with EBV-DNAemia, and whether other potential biomarkers can be used to assess the risk of PTLD developing in pediatric liver transplant patients [77,81].

## Figures and Tables

**Table 1:** EBV detection methods.

Test	Detection Method	Tissue	Biomarker
Antibody Serology	Detects antibodies to viral components of EBV and helps indicate temporality of infection.	Whole blood	*Acute infection:* Anti-VCA IgM, Anti-EA *Post-acute infection:* Anti-EBNA *Acute infection and lifetime persistence:* Anti-VCA IgG
NAAT (PCR)	Detects and quantifies EBV DNAemia through nucleic acid amplification of peripheral blood.	Whole blood or plasma	EBV DNA
EBER Flow FISH	Uses fluorescence in situ hybridization to detect EBV-encoded small RNAs (EBERs). Can determine lytic vs. latent stages and differentiate EBV infections across various cell types.	Tissue or blood	EBV-encoded small RNAs (EBERs)

**Table 2:** WHO 2017 classification of PTLD vs. 2022 International Consensus Classification (ICC) of PTLD.

2017 WHO Classification	2022 International Consensus Classification (ICC) *
Non-destructive PTLD	Hyperplasias arising during immune deficiency/dysregulation
Polymorphic PTLD	Polymorphic lymphoproliferative disorders arising during immune deficiency/dysregulation
Monomorphic PTLD, classic Hodgkin lymphoma PTLD	Lymphomas arising during immune deficiency/dysregulation

* Under the overarching category “Lymphoid proliferations and lymphomas associated with immune deficiency and dysregulation”, which encompasses other diseases outside of PTLD that are not included here.

## Data Availability

No new data were created or analyzed in this study. Data sharing is not applicable to this paper.

## References

[1] de-Thé G., Day N.E., Geser A., Lavoué M.F., Ho J.H., Simons M.J., Sohier R., Tukei P., Vonka V., Zavadova H. (1975). Sero-epidemiology of the Epstein-Barr virus: Preliminary analysis of an international study—A review. IARC Sci. Publ..

[2] Sausen D.G., Bhutta M.S., Gallo E.S., Dahari H., Borenstein R. (2021). Stress-Induced Epstein-Barr Virus Reactivation. Biomolecules.

[3] Dunmire S.K., Verghese P.S., Balfour H.H. (2018). Primary Epstein-Barr virus infection. J. Clin. Virol..

[4] Ou Y., Zhu J., Hou X., Shen X., Xu W., Dong Q., Tan L., Yu J. (2020). Associations of Infectious Agents with Alzheimer’s Disease: A Systematic Review and Meta-Analysis. J. Alzheimer’s Dis..

[5] Houen G., Trier N.H. (2021). Epstein-Barr Virus and Systemic Autoimmune Diseases. Front. Immunol..

[6] Ascherio A., Munger K.L., Lünemann J.D. (2012). The initiation and prevention of multiple sclerosis. Nat. Rev. Neurol..

[7] Rosdahl N., Madsen M., Storm H.H., Askling J., Hamilton-Dutoit S., Melbye M., Frisch M., Helle B.K., Rostgaard K., Zhang J.S. (2003). Characteristics of Hodgkin’s Lymphoma after Infectious Mononucleosis. N. Engl. J. Med..

[8] Gross A., David A. (2004). Thorley-Lawson Persistence of the Epstein–Barr Virus and the Origins of Associated Lymphomas. N. Engl. J. Med..

[9] Haque T., Amlot P.L., Helling N., Thomas J.A., Sweny P., Rolles K., Burroughs A.K., Prentice H.G., Crawford D.H. (1998). Reconstitution of EBV-specific T cell immunity in solid organ transplant recipients. J. Immunol..

[10] Marques-Piubelli M.L., Salas Y.I., Pachas C., Becker-Hecker R., Vega F., Miranda R.N. (2020). Epstein-Barr virus-associated B-cell lymphoproliferative disorders and lymphomas: A review. Pathology.

[11] Dharnidharka V.R. (2018). Comprehensive review of post-organ transplant hematologic cancers. Am. J. Transplant..

[12] Holmes R.D., Sokol R.J. (2002). Epstein-Barr virus and post-transplant lymphoproliferative disease. Pediatr. Transplant..

[13] Okamoto T., Okajima H., Uebayashi E.Y., Ogawa E., Yamada Y., Umeda K., Hiramatsu H., Hatano E. (2022). Management of Epstein–Barr Virus Infection and Post-Transplant Lymphoproliferative Disorder in Pediatric Liver Transplantation. JCM.

[14] Jain A., Nalesnik M., Reyes J., Pokharna R., Mazariegos G., Green M., Eghtesad B., Marsh W., Cacciarelli T., Fontes P. (2002). Posttransplant lymphoproliferative disorders in liver transplantation: A 20-year experience. Ann. Surg..

[15] Quintero Bernabeu J., Juamperez J., Mercadal-Hally M., Larrarte King M., Gallego Melcon S., Gros Subias L., Sábado Álvarez C., Soler-Palacin P., Melendo Pérez S., Esperalba J. (2022). Epstein-Barr virus-associated risk factors for post-transplant lymphoproliferative disease in pediatric liver transplant recipients. Pediatr. Transplant..

[16] Yu H., Robertson E.S. (2023). Epstein–Barr Virus History and Pathogenesis. Viruses.

[17] Paudel S., Lee N. (2024). Epstein-Barr virus noncoding RNA EBER1 promotes the expression of a ribosomal protein paralog to boost oxidative phosphorylation. bioRxiv.

[18] Cen H., Williams P.A., McWilliams H.P., Breinig M.C., Ho M., McKnight J.L. (1993). Evidence for restricted Epstein-Barr virus latent gene expression and anti-EBNA antibody response in solid organ transplant recipients with posttransplant lymphoproliferative disorders. Blood.

[19] Hopwood P., Crawford D.H. (2000). The role of EBV in post-transplant malignancies: A review. J. Clin. Pathol..

[20] Yamada M., Fukuda A., Ogura M., Shimizu S., Uchida H., Yanagi Y., Ishikawa Y., Sakamoto S., Kasahara M., Imadome K. (2023). Early Detection of Epstein-Barr Virus as a Risk Factor for Chronic High Epstein-Barr Viral Load Carriage at a Living-donor-dominant Pediatric Liver Transplantation Center. Transplantation.

[21] Preiksaitis J., Allen U., Bollard C.M., Dharnidharka V.R., Dulek D.E., Green M., Martinez O.M., Metes D.M., Michaels M.G., Smets F. (2024). The IPTA Nashville Consensus Conference on Post-Transplant lymphoproliferative disorders after solid organ transplantation in children: III—Consensus guidelines for Epstein-Barr virus load and other biomarker monitoring. Pediatr. Transplant..

[22] Tomomasa D., Tanita K., Hiruma Y., Hoshino A., Kudo K., Azumi S., Shiota M., Yamaoka M., Eguchi K., Ishimura M. (2024). Highly sensitive detection of Epstein-Barr virus-infected cells by EBER flow FISH. Int. J. Hematol..

[23] Kawabe S., Ito Y., Gotoh K., Kojima S., Matsumoto K., Kinoshita T., Iwata S., Nishiyama Y., Kimura H. (2012). Application of flow cytometric in situ hybridization assay to Epstein-Barr virus-associated T/natural killer cell lymphoproliferative diseases. Cancer Sci..

[24] Su H., Shu Y., Fu G., Liu Z., Zhu D., Zeng L., Ma D., Zou L. (2022). Application of Flow Cytometry Combined Fluorescence in Situ Hybridization to Indentify the Lymphocyte Subtypies with Epstein-Barr Virus Infection. Zhongguo Shi Yan Xue Ye Xue Za Zhi.

[25] Fournier B., Boutboul D., Bruneau J., Miot C., Boulanger C., Malphettes M., Pellier I., Dunogué B., Terrier B., Suarez F. (2020). Rapid identification and characterization of infected cells in blood during chronic active Epstein-Barr virus infection. J. Exp. Med..

[26] Gross T.G., Rubinstein J.D. (2023). Post-transplant lymphoproliferative disease in children, adolescents, and young adults. Hematol. Oncol..

[27] Mynarek M., Schober T., Behrends U., Maecker-Kolhoff B. (2013). Posttransplant lymphoproliferative disease after pediatric solid organ transplantation. Clin. Dev. Immunol..

[28] Collins M.H., Montone K.T., Leahey A.M., Hodinka R.L., Salhany K.E., Kramer D.L., Deng C., Tomaszewski J.E. (2001). Post-transplant lymphoproliferative disease in children. Pediatr. Transplant..

[29] van Esser J.W.J., Niesters H.G.M., van der Holt B., Meijer E., Osterhaus A.D.M.E., Gratama J.W., Verdonck L.F., Löwenberg B., Cornelissen J.J. (2002). Prevention of Epstein-Barr virus-lymphoproliferative disease by molecular monitoring and preemptive rituximab in high-risk patients after allogeneic stem cell transplantation. Blood.

[30] Dike P.N., Schady D., Himes R., Goss J.A., Guffey D., Cerminara D., Mysore K.R. (2024). Incidence and risk factors for chronic rejection in pediatric liver transplantation. Liver Transpl..

[31] Roberts J., Powell J., Mather M.W., Powell S., Brodlie M. (2018). A review of adenotonsillar hypertrophy and adenotonsillectomy in children after solid organ transplantation. Int. J. Pediatr. Otorhinolaryngol..

[32] Pickhardt P.J., Siegel M.J., Hayashi R.J., Kelly M. (2000). Posttransplantation lymphoproliferative disorder in children: Clinical, histopathologic, and imaging features. Radiology.

[33] Akbas A., Tiede C., Lemound J., Maecker-Kolhoff B., Kreipe H., Hussein K. (2015). Post-transplant lymphoproliferative disorders with naso- and oropharyngeal manifestation. Transpl. Int..

[34] Reiche W., Tauseef A., Sabri A., Mirza M., Cantu D., Silberstein P., Chandan S. (2022). Gastrointestinal manifestations, risk factors, and management in patients with post-transplant lymphoproliferative disorder: A systematic review. World J. Transplant..

[35] Rubinstein J., Toner K., Gross T., Wistinghausen B. (2023). Diagnosis and management of post-transplant lymphoproliferative disease following solid organ transplantation in children, adolescents, and young adults. Best. Pract. Res. Clin. Haematol..

[36] Hoyt D., Hughes J., Liu J., Ayyad H. (2024). Primary central nervous system post-transplantation lymphoproliferative disorder: A case report and systematic review of imaging findings. Radiol. Case Rep..

[37] Taj M.M., Maecker-Kolhoff B., Ling R., Bomken S., Burkhardt B., Chiang A.K.S., Csoka M., Füreder A., Haouy S., Lazic J. (2021). Primary post-transplant lymphoproliferative disorder of the central nervous system: Characteristics, management and outcome in 25 paediatric patients. Br. J. Haematol..

[38] Cohen J.I. (2000). Epstein-Barr virus infection. N. Engl. J. Med..

[39] Montanari F., Orjuela-Grimm M. (2021). Joining Efforts for PTLD: Lessons Learned from Comparing the Approach and Treatment Strategies Across the Pediatric and Adult Age Spectra. Curr. Hematol. Malig. Rep..

[40] Schober T., Framke T., Kreipe H., Schulz T.F., Großhennig A., Hussein K., Baumann U., Pape L., Schubert S., Wingen A. (2013). Characteristics of early and late PTLD development in pediatric solid organ transplant recipients. Transplantation.

[41] Swerdlow S.H., Campo E., Harris N.L., Jaffe E.S., Pileri S.A., Stein H., Thiele J., Vardiman J.W. (2008). WHO Classification of Tumours of Haematopoietic and Lymphoid Tissues.

[42] Alaggio R., Amador C., Anagnostopoulos I., Attygalle A.D., Araujo I.B.d.O., Berti E., Bhagat G., Borges A.M., Boyer D., Calaminici M. (2022). The 5th edition of the World Health Organization Classification of Haematolymphoid Tumours: Lymphoid Neoplasms. Leukemia.

[43] Lauro A., Arpinati M., Pinna A.D. (2015). Managing the challenge of PTLD in liver and bowel transplant recipients. Br. J. Haematol..

[44] Campo E., Jaffe E.S., Cook J.R., Quintanilla-Martinez L., Swerdlow S.H., Anderson K.C., Brousset P., Cerroni L., de Leval L., Dirnhofer S. (2022). The International Consensus Classification of Mature Lymphoid Neoplasms: A report from the Clinical Advisory Committee. Blood.

[45] DeStefano C.B., Desai S.H., Shenoy A.G., Catlett J.P. (2018). Management of post-transplant lymphoproliferative disorders. Br. J. Haematol..

[46] Cheson B.D., Fisher R.I., Barrington S.F., Cavalli F., Schwartz L.H., Zucca E., Lister T.A. (2014). Recommendations for Initial Evaluation, Staging, and Response Assessment of Hodgkin and Non-Hodgkin Lymphoma: The Lugano Classification. J. Clin. Oncol..

[47] L’Huillier A.G., Dipchand A.I., Ng V.L., Hebert D., Avitzur Y., Solomon M., Ngan B., Stephens D., Punnett A.S., Barton M. (2019). Posttransplant lymphoproliferative disorder in pediatric patients: Survival rates according to primary sites of occurrence and a proposed clinical categorization. Am. J. Transplant..

[48] Allen U.D., Preiksaitis J.K., AST Infectious Diseases Community of Practice (2019). Post-transplant lymphoproliferative disorders, Epstein-Barr virus infection, and disease in solid organ transplantation: Guidelines from the American Society of Transplantation Infectious Diseases Community of Practice. Clin. Transplant..

[49] Tajima T., Martinez O.M., Bernstein D., Boyd S.D., Gratzinger D., Lum G., Sasaki K., Tan B., Twist C.J., Weinberg K. (2024). Epstein-Barr virus-associated post-transplant lymphoproliferative disorders in pediatric transplantation: A prospective multicenter study in the United States. Pediatr. Transplant..

[50] Liu J., Gao H., Xu L., Mo X., Liu R., Liang S., Wu N., Wang M., Wang Z., Chang Y. (2020). Immunosuppressant indulges EBV reactivation and related lymphoproliferative disease by inhibiting Vδ2(+) T cells activities after hematopoietic transplantation for blood malignancies. J. Immunother. Cancer.

[51] Xiang Z., Liu Y., Zheng J., Liu M., Lv A., Gao Y., Hu H., Lam K., Chan G.C., Yang Y. (2014). Targeted activation of human Vγ9Vδ2-T cells controls epstein-barr virus-induced B cell lymphoproliferative disease. Cancer Cell.

[52] Hatayama Y., Hashimoto Y., Motokura T. (2020). Frequent co-reactivation of Epstein-Barr virus in patients with cytomegalovirus viremia under immunosuppressive therapy and/or chemotherapy. J. Int. Med. Res..

[53] Kerr B.M., Lear A.L., Rowe M., Croom-Carter D., Young L.S., Rookes S.M., Gallimore P.H., Rickinson A.B. (1992). Three transcriptionally distinct forms of Epstein-Barr virus latency in somatic cell hybrids: Cell phenotype dependence of virus promoter usage. Virology.

[54] Shannon-Lowe C., Rickinson A.B., Bell A.I. (2017). Epstein-Barr virus-associated lymphomas. Philos. Trans. R. Soc. Lond. B Biol. Sci..

[55] Yamada M., Macedo C., Louis K., Shi T., Landsittel D., Nguyen C., Shinjoh M., Michaels M.G., Feingold B., Mazariegos G.V. (2023). Distinct association between chronic Epstein-Barr virus infection and T cell compartments from pediatric heart, kidney, and liver transplant recipients. Am. J. Transplant..

[56] Dharnidharka V.R., Lamb K.E., Gregg J.A., Meier-Kriesche H. (2012). Associations between EBV serostatus and organ transplant type in PTLD risk: An analysis of the SRTR National Registry Data in the United States. Am. J. Transplant..

[57] Ferla V., Rossi F.G., Goldaniga M.C., Baldini L. (2020). Biological Difference Between Epstein-Barr Virus Positive and Negative Post-transplant Lymphoproliferative Disorders and Their Clinical Impact. Front. Oncol..

[58] Morscio J., Dierickx D., Tousseyn T. (2013). Molecular pathogenesis of B-cell posttransplant lymphoproliferative disorder: What do we know so far?. Clin. Dev. Immunol..

[59] Hurwitz M., Desai D.M., Cox K.L., Berquist W.E., Esquivel C.O., Millan M.T. (2004). Complete immunosuppressive withdrawal as a uniform approach to post-transplant lymphoproliferative disease in pediatric liver transplantation. Pediatr. Transplant..

[60] Al Hamed R., Bazarbachi A.H., Mohty M. (2020). Epstein-Barr virus-related post-transplant lymphoproliferative disease (EBV-PTLD) in the setting of allogeneic stem cell transplantation: A comprehensive review from pathogenesis to forthcoming treatment modalities. Bone Marrow Transplant..

[61] Allen U.D., L’Huillier A.G., Bollard C.M., Gross T.G., Hayashi R.J., Höcker B., Maecker-Kolhoff B., Marks S.D., Mazariegos G.V., Smets F. (2024). The IPTA Nashville consensus conference on post-transplant lymphoproliferative disorders after solid organ transplantation in children: IV-consensus guidelines for the management of post-transplant lymphoproliferative disorders in children and adolescents. Pediatr. Transplant..

[62] Al-Mansour Z., Nelson B.P., Evens A.M. (2013). Post-transplant lymphoproliferative disease (PTLD): Risk factors, diagnosis, and current treatment strategies. Curr. Hematol. Malig. Rep..

[63] Choquet S., Leblond V., Herbrecht R., Socié G., Stoppa A., Vandenberghe P., Fischer A., Morschhauser F., Salles G., Feremans W. (2006). Efficacy and safety of rituximab in B-cell post-transplantation lymphoproliferative disorders: Results of a prospective multicenter phase 2 study. Blood.

[64] Oertel S.H.K., Verschuuren E., Reinke P., Zeidler K., Papp-Váry M., Babel N., Trappe R.U., Jonas S., Hummel M., Anagnostopoulos I. (2005). Effect of anti-CD 20 antibody rituximab in patients with post-transplant lymphoproliferative disorder (PTLD). Am. J. Transplant..

[65] Evens A.M., David K.A., Helenowski I., Nelson B., Kaufman D., Kircher S.M., Gimelfarb A., Hattersley E., Mauro L.A., Jovanovic B. (2010). Multicenter analysis of 80 solid organ transplantation recipients with post-transplantation lymphoproliferative disease: Outcomes and prognostic factors in the modern era. J. Clin. Oncol..

[66] Trappe R.U., Dierickx D., Zimmermann H., Morschhauser F., Mollee P., Zaucha J.M., Dreyling M.H., Dührsen U., Reinke P., Verhoef G. (2017). Response to Rituximab Induction Is a Predictive Marker in B-Cell Post-Transplant Lymphoproliferative Disorder and Allows Successful Stratification Into Rituximab or R-CHOP Consolidation in an International, Prospective, Multicenter Phase II Trial. J. Clin. Oncol..

[67] Trappe R., Oertel S., Leblond V., Mollee P., Sender M., Reinke P., Neuhaus R., Lehmkuhl H., Horst H.A., Salles G. (2012). Sequential treatment with rituximab followed by CHOP chemotherapy in adult B-cell post-transplant lymphoproliferative disorder (PTLD): The prospective international multicentre phase 2 PTLD-1 trial. Lancet Oncol..

[68] Gross T.G., Orjuela M.A., Perkins S.L., Park J.R., Lynch J.C., Cairo M.S., Smith L.M., Hayashi R.J. (2012). Low-dose chemotherapy and rituximab for posttransplant lymphoproliferative disease (PTLD): A Children’s Oncology Group Report. Am. J. Transplant..

[69] Vase M.Ø., Maksten E.F., Bendix K., Hamilton-Dutoit S., Andersen C., Møller M.B., Sørensen S.S., Jespersen B., Kampmann J., Søndergård E. (2015). Occurrence and prognostic relevance of CD30 expression in post-transplant lymphoproliferative disorders. Leuk. Lymphoma.

[70] Markouli M., Ullah F., Omar N., Apostolopoulou A., Dhillon P., Diamantopoulos P., Dower J., Gurnari C., Ahmed S., Dima D. (2022). Recent Advances in Adult Post-Transplant Lymphoproliferative Disorder. Cancers.

[71] Pearse W.B., Petrich A.M., Gordon L.I., Karmali R., Winter J.N., Ma S., Kaplan J.B., Behdad A., Klein A., Jovanovic B. (2021). A phase I/II trial of brentuximab vedotin plus rituximab as frontline therapy for patients with immunosuppression-associated CD30+ and/or EBV + lymphomas. Leuk. Lymphoma.

[72] Haque T., Wilkie G.M., Jones M.M., Higgins C.D., Urquhart G., Wingate P., Burns D., McAulay K., Turner M., Bellamy C. (2007). Allogeneic cytotoxic T-cell therapy for EBV-positive posttransplantation lymphoproliferative disease: Results of a phase 2 multicenter clinical trial. Blood.

[73] Mahadeo K.M., Baiocchi R., Beitinjaneh A., Chaganti S., Choquet S., Dierickx D., Dinavahi R., Duan X., Gamelin L., Ghobadi A. (2024). Tabelecleucel for allogeneic haematopoietic stem-cell or solid organ transplant recipients with Epstein-Barr virus-positive post-transplant lymphoproliferative disease after failure of rituximab or rituximab and chemotherapy (ALLELE): A phase 3, multicentre, open-label trial. Lancet Oncol..

[74] Haddad E., Paczesny S., Leblond V., Seigneurin J.M., Stern M., Achkar A., Bauwens M., Delwail V., Debray D., Duvoux C. (2001). Treatment of B-lymphoproliferative disorder with a monoclonal anti-interleukin-6 antibody in 12 patients: A multicenter phase 1-2 clinical trial. Blood.

[75] Simmons H.Z., Bazzell A.F., Dains J.E. (2019). Adverse Effects of Virus-Specific T-Cell Therapy: An Integrative Review. J. Adv. Pract. Oncol..

[76] Campbell L., Chen C., Bhagat S.S., Parker R.A., Östör A.J.K. (2011). Risk of adverse events including serious infections in rheumatoid arthritis patients treated with tocilizumab: A systematic literature review and meta-analysis of randomized controlled trials. Rheumatology.

[77] Yamada M., Chen S.F., Green M. (2024). Chronic Epstein-Barr viral load carriage after pediatric organ transplantation. Front. Pediatr..

[78] Chen H., Ho M., Hu R., Wu J., Chen H., Ni Y., Hsu H., Jeng Y., Chang M. (2019). Roles of Epstein-Barr virus viral load monitoring in the prediction of posttransplant lymphoproliferative disorder in pediatric liver transplantation. J. Formos. Med. Assoc..

[79] Seo E., Kim J., Oh S.H., Kim K.M., Kim D.Y., Lee J. (2020). Epstein-Barr viral load monitoring for diagnosing post-transplant lymphoproliferative disorder in pediatric liver transplant recipients. Pediatr. Transplant..

[80] Green M., Squires J.E., Chinnock R.E., Comoli P., Danziger-Isakov L., Dulek D.E., Esquivel C.O., Höcker B., L’Huillier A.G., Mazariegos G.V. (2024). The IPTA Nashville consensus conference on Post-Transplant lymphoproliferative disorders after solid organ transplantation in children: II—Consensus guidelines for prevention. Pediatr. Transplant..

[81] Baker A., Frauca Remacha E., Torres Canizales J., Bravo-Gallego L.Y., Fitzpatrick E., Alonso Melgar A., Muñoz Bartolo G., Garcia Guereta L., Ramos Boluda E., Mozo Y. (2021). Current Practices on Diagnosis, Prevention and Treatment of Post-Transplant Lymphoproliferative Disorder in Pediatric Patients after Solid Organ Transplantation: Results of ERN TransplantChild Healthcare Working Group Survey. Children.

